# The association of dietary nitrates/nitrites intake and the gut microbial metabolite trimethylamine N-oxide and kynurenine in adults: a population-based study

**DOI:** 10.3389/fnut.2024.1346074

**Published:** 2024-02-21

**Authors:** Atieh Mirzababaei, Maryam Mahmoodi, Faezeh Abaj, Bahareh Barkhidarian, Azadeh Dehghani, Pardis Khalili, Zahra Roumi, Khadijeh Mirzaei

**Affiliations:** ^1^Department of Community Nutrition, School of Nutritional Sciences and Dietetics, Tehran University of Medical Sciences, Tehran, Iran; ^2^Department of Cellular and Molecular Nutrition, School of Nutritional Science and Dietetics, Tehran University of Medical Sciences, Tehran, Iran; ^3^Department of Nutrition, Dietetics and Food, School of Clinical Sciences at Monash Health, Monash University, Clayton, VIC, Australia; ^4^Department of Community Nutrition, Faculty of Nutrition and Food Science, Nutrition Research Center, Tabriz University of Medical Sciences, Tabriz, Iran; ^5^Department of Nutrition, Science and Research Branch, Islamic Azad University, Tehran, Iran

**Keywords:** dietary nitrates, nitrites, gut microbial metabolite, trimethylamine N-oxide kynurenine, nitric oxide

## Abstract

**Background:**

Dietary nitrate and nitrite may affect the gut microbiota and its metabolites, such as trimethylamine N-oxide (TMAO) and kynurenine (KYN). However, this association and the exact mechanism are still unclear. Therefore, this study aimed to assess the association between dietary consumption of nitrite and nitrate on TMAO and KYN levels in adults.

**Methods:**

This cross-sectional study was employed on a subsample baseline phase of the Tehran University of Medical Sciences (TUMS) Employee's Cohort Study (TEC). A total of 250 adults aged 18 years or older were included in the current analysis. Data on the dietary intakes were collected using a validated dish-based food frequency questionnaire (FFQ), and dietary intakes of nitrite and nitrate were estimated using the FFQ with 144 items. Serum profiles and TMAO and KYN were measured using a standard protocol.

**Results:**

The findings of this study demonstrate a significant association between the intake of animal sources of nitrate and nitrite and the likelihood of having elevated levels of TMAO and KYN. Specifically, after adjustment, individuals with the highest intake adherence to nitrates from animal sources exhibited increased odds of having the highest level of TMAO (≥51.02 pg/ml) (OR = 1.51, 95% CI = 0.59–3.88, *P* = 0.03) and KYN (≥417.41 pg/ml) (OR = 1.75, 95% CI = 0.73–4.17, *P* = 0.02). Additionally, subjects with the highest animal intake from nitrite sources have 1.73 and 1.45 times higher odds of having the highest levels of TMAO and KYN. These results emphasize the potential implications of animal-derived nitrate and nitrite consumption on the levels of TMAO and KYN.

**Conclusion:**

The present evidence indicates that a high level of nitrate and nitrite intake from animal sources can increase the odds of high levels of TMAO and KYN. Further studies suggest that we should better evaluate and understand this association.

## 1 Introduction

Ongoing studies have determined the crucial role played by the gut microbiota in human health and disease (1). The gut microbiota comprises trillions of microorganisms, including bacteria, residing within the gastrointestinal tract (2, 3), and may influence metabolic, endocrine, immune, and cardiovascular activity (4–8). Dysbiosis, an alteration in the gut microbiota, occurs with changes in the quantity, diversity, or function of the gut microbiota and is associated with human health (9). Many factors may affect the gut microbiota and its metabolites (10–12). Dietary components, mainly nitrates and nitrates, have attracted huge attention due to their occurrence in specific foods and their potential effect on gut microbiota metabolites. Dietary exposure to nitrate and nitrite occurs through fruits, vegetables, and their derivatives; processed foods containing nitrate and nitrite salts as food additives; and drinking water (13). Moreover, nitrate undergoes endogenous conversion to nitrite within the human body via enzymatic reactions or oral microbial activity. This conversion process, indicated by an increase in nitrate-reducing bacteria, is known to influence the composition and potentially the metabolic capacity of the oral microbiota. Therefore, it elevates plasma and salivary nitrite levels, leading to cardiovascular protection (14–16). However, the effect of dietary nitrate on the gut microbiota and its metabolites remains largely elusive. One of the most important metabolites produced by the gut microbiota is trimethylamine N-oxide (TMAO), which may affect human health, particularly cardiovascular health (17). The gut microbiota metabolizes dietary components, including choline, carnitine, phosphatidylcholine, and betaine, to produce trimethylamine (TMA). This TMA is then oxidized in the liver by the flavin-containing monooxygenase 3 (FMO3) enzyme to form TMAO (18, 19).

There are some possible mechanisms linking dietary nitrate and nitrite to TMAO status. First, dietary nitrate and nitrite intake may affect the composition and function of the intestinal microbiota (20, 21), inhibit the growth of TMA-producing bacteria, or compete with TMA precursors for microbial metabolism, resulting in a lower level of TMAO production. However, inconsistent findings regarding the effect of a mixture of fruits and vegetables, or nitrate, on microbiota were observed in the few studies (20–23). Second, nitrate and nitrate can be converted into nitric oxide (NO) by enzymatic mechanisms (24). The gut microbiota's composition and function are impacted by NO (25). It has antibacterial qualities (26) and can prevent the growth and survival of some types of intestinal bacteria. Therefore, changes in the composition of the gut microbiota may have an impact on TMAO levels. Through its impact on TMA metabolism, NO can also indirectly affect TMAO levels. The enzyme FMO3, which transforms TMA into TMAO in the liver, can be inhibited by NO (27, 28). By inhibiting FMO3, NO can reduce the production of TMAO. Furthermore, the gut barrier function is regulated by NO (29). It might help the integrity of the gut barrier (30), which prevents the translocation of bacteria and their metabolites into systemic circulation. Impaired gut barrier function can contribute to increased bacterial translocation and the release of TMA and other bacterial metabolites into the bloodstream, potentially influencing TMAO levels (29).

Kynurenine (KYN) is another important factor, which is a tryptophan metabolite. KYN plays an important role in immune regulation (31) and in neurological functions (32, 33). KYN is produced through a series of enzymatic reactions known as the kynurenine pathway (34). It can have direct and indirect effects on gut bacteria (32). Dietary nitrates and nitrates may alter KYN levels through several potential mechanisms. Nitrate and nitrite may affect KYN production through conversion to NO. NO regulates indoleamine 2,3-dioxygenases (IDO), a tryptophan-degrading enzyme, which is involved in tryptophan metabolism and KYN production (35). Additionally, the gut microbiota, affected by dietary nitrate and nitrite (20, 21), may impact KYN levels through enzymatic activity and regulation of tryptophan availability (32). However, it should be noted that despite all the mentioned mechanisms, the precise means linking dietary nitrate and nitrate to TMAO and KYN metabolism still need to be researched.

Therefore, because the studies in this field are few and limited, we aimed to assess the association between dietary nitrate and nitrite with TMAO and kynurenine status in the adult population. The result of our investigation may help better understand the possible role of dietary nitrate and nitrite in regulating gut microbiota metabolism and its later effect on human health.

## 2 Methods and materials

### 2.1 Participants

This cross-sectional study was carried out between 2018 and 2020 on a subsample baseline phase of subjects from the Tehran University of Medical Sciences (TUMS) Employee's Cohort Study (TEC), who were aged between 20 years and 50 years. The TUMS Ethics Committee approved the study protocol (IR.TUMS.MEDICINE.REC.1401.064), and all subjects gave their consent before participation (36). Exclusion criteria included those with acute or chronic diseases (including diabetes mellitus, infectious diseases, intestinal disorders, cancers, PCOS, and hepatic and kidney diseases), alcohol consumption, pregnancy or lactation, adherence to special diets, significant body weight changes in the previous year, or use of medications affecting body weight, glucose, lipid levels, or probiotics and antibiotics. Subjects who consumed < 800 or >4,200 (kcal/day) were also excluded.

### 2.2 Assessment of dietary intakes

The dietary information of individuals was assessed using a validated and reliable 144-item dish-based food frequency questionnaire (FFQ) (37). The questionnaire included questions about various food groups, with nine response options. Daily food intake was analyzed using the NUTRITIONIST 4 Food Analyzer (First Data Bank, San Bruno, CA) and cross-checked with the U.S. Department of Agriculture's National Nutrient Databank (38).

### 2.3 Quantification of dietary nitrate and nitrite

Due to the incomplete nature of the Iranian Food Composition Table, which lacks comprehensive data on the nutrient content of raw foods and beverages, the U.S. Department of Agriculture Food Composition Table (excluding nitrate and nitrite) was employed to analyze the energy and nutrient content of foods and beverages (39). However, for the assessment of nitrate and nitrite levels, specific food composition values were obtained from a recent survey focusing on commonly consumed food items among Iranians. In this study, the nitrate and nitrite contents of the food items were determined based on relevant research conducted in Iran (40).

### 2.4 Biochemical and laboratory assessments

To perform biochemical assays, we collected venous blood samples from each participant between 8:00 AM and 10:00 AM, after a fasting period of 12 h−14 h. All blood samples were centrifuged at 3,000 rpm for 10 min to separate and freeze the serum for further testing. The serum concentrations of kynurenine and TMAO were determined using an ELISA kit (Crystal Day Biotech Co., Ltd.). The biochemical analyses were performed using commercially available kits (Pars Azmoon Inc., Tehran, Iran). Fasting blood sugar (FBS), total cholesterol (TC), low-density lipoprotein (LDL-C), triglyceride (TG), and high-density lipoprotein (HDL-C) levels were measured using an enzymatic colorimetric method and phosphor tungstic acid, respectively.

### 2.5 Anthropometric measurements

Standardized methods were used to evaluate anthropometric measurements. Height was measured with a non-elastic measuring tape, accurate to ~0.1 cm, in a standing position without shoes. Waist circumference (WC) was measured with a non-elastic tape measure, accurate to ~0.1 cm, at the narrowest point between the lower rib margin and iliac crest. Hip circumference (HC) was measured with an inelastic tape measure, accurate to ~0.1 cm, at the widest part of the buttocks. The waist-hip ratio (WHR) was calculated by dividing WC by HC, and the body mass index (BMI) was calculated using the formula weight/height^2^ (kg/m^2^) (41).

### 2.6 Other variables

Physical activity (PA) was assessed using the internationally recognized and validated self-report International Physical Activity Questionnaire (IPAQ). This questionnaire has been widely used both globally and among Iranian adults. PA levels were measured for the past 7 days and expressed as metabolic equivalents (METs) (42). Scores were calculated based on the frequency and duration of light, moderate, high, and very high-intensity activities and then summed to obtain the total MET minutes per week. Additionally, socio-demographic factors such as education, occupation, marital status, smoking status, socioeconomic status (SES), and supplementation intake were assessed through a self-reported socio-demographic questionnaire.

### 2.7 Statistical analysis

The Kolmogorov-Smirnov test (*P* > 0.05) was used to confirm the normality of the data distribution; the data were found to be non-normally distributed. The tables display descriptive data from study participants as frequencies (n) and percentages (%) for categorical variables and mean and standard deviation for continuous parameters.

We classified gut microbial metabolites (TMAO and KYN) into three groups. ANOVA and chi-square tests were used to examine the differences in baseline characteristics and food intakes between the tertiles of levels TMAO and KYN. Furthermore, we used ANCOVA to consider confounders such as age, gender, energy intake, and physical activity in the adjusted model.

The significance of the association between plasma levels of TMAO and KYN and selected food items (dietary total nitrate and nitrite, as well as plant and animal sources) was evaluated using a multinominal regression model. In this model, the first tertile of both dietary nitrate and nitrite intake and plasma levels of TMAO and KYN were considered as reference groups. The regression model was adjusted for age, gender, total energy, BMI, and physical activity. Statistical analysis was carried out utilizing SPSS software version 25. For all analyses, P < 0.05 was considered significant.

## 3 Results

### 3.1 Study population characteristics

This comparative cross-sectional study was conducted on 250 participants, 26% of whom were men. The means and standard deviation (SD) of age, WC, BMI, SBP and DBP, and BMI were, respectively, 41.27 (8.7) years, 90.63 (11.53) cm, 27.24 (4.48) kg/m^2^, 116.16 (17.44) mmHg, and 76.80 (11.67) mmHg. The current study was on a cohort of university employees, of whom 29.9% were office workers and 32.8% were health service workers. In total, 90% had average socioeconomic status, 42% were smokers, and 80% of these participants were married. The mean levels of TMAO and KYN in subjects were 44.49 (36.75) pg/ml and 393.54 (284.39) nmol/L, respectively. The mean total intake of nitrate and nitrite was 672.77 (631.69) mg/day and 12.57 (12.18) mg/day; the mean intake of nitrate and nitrite of plant sources was 648.66 (238.35) mg/day and 5.96 (1.93) mg/day observed; and the mean intake of nitrate and nitrite of animal sources was 24.10 (10.40) mg/day and 6.74 (2.96) mg/day.

### 3.2 Characteristics of participants among the levels of TMAO and KYN tertiles

The characteristics of participants are compared across tertile levels of TMAO and KYN in Table 1. After adjusting for confounders (age, sex, physical activity, BMI, and total energy intake), we observed a significant increase between the levels of TMAO tertiles and average WC, HC, TG, and FBS.

**Table 1:** Characteristics of the participants among levels of TMAO and KYN tertiles.

**Variables**	**TMAO (pg/ml)**					**KYN (nmol/l)**				
	**T1**	**T2**	**T3**			**T1**	**T2**	**T3**		
	**(** ≤ **23.77)**	**(23.77-51.02)**	**(**≥**51.02)**	***P-*** **value**	***P-*** **value** ^*^	**(** ≤ **225.35)**	**(225.35-417.41)**	**(**≥**417.41)**	***P-*** **value**	***P-*****value** ^*^
	***n** =* **80**	***n** =* **78**	***n** =* **92**			***n** =* **86**	***n** =* **75**	***n** =* **89**		
**Demographic**										
Age (year)	41.35 (8.95)	41.30 (8.98)	41.14 (8.23)	0.98	0.99	41.19 (8.65)	40.77 (9.52)	41.83 (7.96)	0.75	0.99
**Anthropometric**										
Weight (kg)	75.4 (13.65)	74.12 (15.77)	27.76 (13.97)	0.52	0.98	75.14 (13.27)	73.53 (15.74)	73.60 (14.54)	0.73	0.9
Height (cm)	165.51 (9.17)	164.55 (8.51)	164.09 (8.80)	0.58	0.94	165.76 (9.58)	163.15 (8.24)	165.17 (8.41)	0.15	0.64
BMI (kg/m^2^)	27.48 (4.25)	27.24 (4.56)	26.99 (4.66)	0.79	0.88	27.26 (3.76)	27.15 (4.68)	26.95 (4.99)	0.74	0.89
WHR	0.86 (0.09)	0.85 (0.08)	0.89 (0.08)	0.61	0.95	0.85 (0.08)	0.87 (0.08)	0.88 (0.09)	0.41	0.87
WC (cm)	90.81 (12.55)	87.99 (10.58)	93.1 (10.9)	0.14	**0.01**	87.08 (9.73)	89.67 (11.86)	91.28 (11.22)	**0.03**	< 0.001
HC (cm)	104.05 (9.93)	102.63 (7.59)	107.05 (7.07)	**0.004**	**0.007**	102.02(6.31)	105.43 (7.69)	106.43 (9.75)	**0.002**	0.04
**Blood parameters**										
TG (mg/dl)	146.01 ± 68.18	150.84 ± 99.73	171.17 ± 117.47	0.23	**0.03**	154.79 (111.97)	143.25 (73.72)	169.76 (100.01)	0.24	0.26
HDL (mg/dl)	43.37 (8.07)	44.64 (8.84)	42.98 (8.51)	0.44	0.34	43.2 (8.5)	43.38 (8.13)	44.44 (8.82)	0.61	0.58
LDL (mg/dl)	105.8 (25.73)	105.17 (26.81)	106.06 (29.94)	0.97	0.95	101.08 (30.14)	106.57 (26.76)	109.04 (25.01)	0.18	0.61
TC (mg/dl)	181.12 (33.38)	182.25 (37.15)	189.22 (51.23)	0.41	0.93	183.81 (37.31)	188.76 (39.15)	194.77 (46.87)	0.15	0.06
FBS (mg/dl)	93.61 (27.21)	87.65 (29.95)	101.34 (45.16)	**0.05**	**0.04**	89.31 (21.53)	92.91 (27.09)	100.42 (50.43)	0.13	0.04
**Blood Pressure**										
SBP (mmHg)	117.60 (17.92)	113.31 (15.12)	117.57 (18.94)	0.21	0.55	117.89 (16.58)	114.95 (17.16)	115.49 (18.63)	0.52	0.98
DBP (mmHg)	78.36 (11.6)	75.27 (10.48)	76.72 (12.78)	0.25	0.53	78.17 (11.57)	75.73 (10.92)	76.38 (12.46)	0.39	0.7
**Qualitative variable**										
**Sex**										
Male	26 (30.5)	23 (27)	36 (42.5)			29 (40.3)	17 (25.4)	23 (34.3)		
Female	54 (31.7)	55 (32.8)	56(35.5)	0.39	0.98	57 (31.1)	58 (32.1)	66(36.8)	0.22	0.98
**PA (Mets)**										
Low	76 (32.7)	74 (31.8)	82 (35.5)			83 (35.6)	71 (30.4)	79 (34)		
Moderate	4 (22)	4 (22)	10 (56)	0.35	0.5	3 (17.6)	4 (23.5)	10 (58.9)	0.34	0.61
**Marriage status**										
Single	14 (28.6)	16 (32.7)	19 (38.8)			11 (22.4)	20 (40.8)	18 (36.7)		
Married	66 (32.8)	62 (30.8)	73 (36.4)	0.57	0.45	75 (37.3)	55 (27.3)	71 (35.4)	0.16	0.21
**Education level**										
≤ Diploma	28 (32.6)	28 (32.6)	30 (34.9)			30 (34.9)	29 (33.7)	27 (31.4)		
University education	52 (31.7)	50 (30.3)	62 (38)	0.67	0.71	56 (34.1)	46 (28)	62 (37.9)	0.7	0.68
**Job group**										
Office worker	38 (29.9)	50 (39.9)	39 (30.7)			38 (29.9)	40 (31.5)	49 (38.6)		
Health service	22 (32.8)	20 (29.9)	25 (37.3)	0.01	0.01	28 (41.8)	24 (35.8)	15 (22.4)	0.08	0.1
Technical worker	10 (28.5)	2 (5.5)	23 (66)			4 (26.6)	2 (13)	9 (60.4)		
Guarding worker	10 (47.6)	6 (28.6)	5 (23.8)			16 (39)	9 (22)	16 (39)		
**Socio-economic status**										
Low	4 (40)	2 (20)	4 (40)			4 (40)	2 (20)	4 (40)		
Moderate	70 (31)	72 (32)	84 (37)	0.84	0.83	75 (34.4)	70 (32.1)	73(33.5)	0.74	0.71
High	6 (42.9)	4 (28.6)	4 (28.6)			7 (31.8)	3 (13.6)	12 (54.6)		
**Smoking**										
Yes	39 (36.4)	36 (33.6)	32 (29.9)			29 (27.1)	33 (30.8)	45 (42.1)		
				0.58	0.61				0.04	0.03
No	41 (28.6)	42 (29.3)	60 (42.1)			57 (40)	42 (29.3)	44 (30.7)		

PA, physical activity; BMI, body mass index; HC, hip circumference; WC, waist circumference; WHR, weight to hip ratio; FBS, fasting blood sugar; TC, total cholesterol; TG, triglyceride; HDL, high-density lipoprotein; LDL, low-density lipoprotein. Quantitative variables as means (± SD) obtained from the independent t-test. Qualitative variables N (%) obtained from the chi-square analysis. P < 0.05 were considered significant. P-values^*^ is found by ANCOVA and adjusted for age, sex, physical activity BMI, and total energy intake. Bold values indicate *p* < 0.05.

The results of our study show a significant difference before and after potential confounders between the average WC and HC among tertiles of KYN, so the mean WC in subjects who were in the third tertile of KYN was 91.28 (11.22) cm vs. 87.08 (9.73) cm in the first tertile, and the mean HC was 106.43 (94.75) cm in the third tertile vs. 102.02 (672.31) cm in the first tertile, which showed an increase in trend. Furthermore, the results of this study indicated that there was a significant difference between FBS and TC and levels of KYN (*P* < 0.05) after controlling for confounders.

Among the qualitative variables, a significant difference between job groups was observed among the levels of TMAO and KYN. People who were in the third tertile of KYN (42.1%) had a higher smoking prevalence than those who were in the first tertile (27.1%), and this difference was significant (*P* = 0.04).

### 3.3 Characteristics of participants among tertiles of dietary nitrate and nitrite intake

The characteristics of participants among tertiles of dietary nitrate and nitrite intake are shown in Table 2. The results of this study showed that a significant difference was observed between the average TG and SBP after adjusting for confounders. In subjects with higher intakes of nitrate, the mean of TG and SBP decreased (*P* < 0.05).

**Table 2:** Characteristics of the participants among the intake of tertile nitrate and nitrite.

**Variables**	**Nitrate (mg/day)**					**Nitrite (mg/day)**				
	**T1 (** ≤ **540.58)** ***n** =* **83**	**T2 (540.58-750.23)** ***n** =* **77**	**T3 (**≥**750.23)** ***n** =* **90**	***P*** **-value**	***P*****-value** ^*^	**T1 (** ≤ **10.62)** ***n** =* **87**	**T2 (10.62-13.87)** ***n** =* **89**	**T3 (**≥**13.87)** ***n** =* **74**	***P*** **-value**	***P*****-value** ^*^
**Demographic**										
Age (year)	41.31 (8.72)	42.35 (8.24)	41.18 (9.15)	0.69	0.9	41.30 (8.57)	41.46 (8.6)	42.2 (8.88)	0.81	0.99
**Anthropometric**										
Weight (kg)	73.49 (14.26)	73.99 (13.71)	73.78 (13.68)	0.97	0.98	73.87 (14.82)	73.11 (13.38)	74.15 (13.23)	0.91	0.99
Height (cm)	165.59 (8.9)	163.17 (7.64)	164.91 (8.98)	0.21	0.53	165.82 (8.62)	162.86 (8.31)	164.58 (8.47)	0.13	0.63
BMI (kg/m^2^)	26.72 (4.34)	27.77 (4.78)	26.99 (3.62)	0.32	0.88	26.73 (4.39)	27.52 (4.37)	27.34 (4.24)	0.53	0.69
WHR	0.84 (0.07)	0.86 (0.08)	0.85 (0.08)	0.36	0.88	0.85 (0.08)	0.84 (0.08)	0.86 (0.08)	0.54	0.72
WC (cm)	88.43 (10.19)	89.49 (11.94)	90.08 (10.99)	0.68	0.62	89.32 (10.97)	88.17 (10.25)	90.09 (11.17)	0.62	0.64
HC (cm)	104.68 (8.63)	103.46 (8.94)	105.44 (7.31)	0.88	0.66	104.3 (8.86)	104.47 (7.85)	104.65 (8.46)	0.97	0.51
**Blood parameters**										
TG (mg/dl)	153.54 (85.42)	160.94 (102.88)	146.07 (79.26)	0.16	0.04	157.75 (85.97)	144.12 (77.87)	158.66 (104.72)	0.6	0.39
HDL (mg/dl)	42.97 (7.99)	43.68 (8.08)	43.38 (8.98)	0.87	0.33	42.21 (8.31)	44.6 (8.94)	43.53 (7.48)	0.24	0.7
LDL (mg/dl)	107.36 (28.58)	103.8 (26.93)	101.88 (26.96)	0.51	0.96	106.14 (28.14)	104.68 (23.26)	102.83 (30.49)	0.17	0.03
TC (mg/dl)	186.07 (48.17)	181.57 (34.85)	178.11 (38.23)	0.54	0.95	184.37 (47.62)	180.65 (31.8)	181.49 (40.87)	0.85	0.94
FBS (mg/dl)	95.97 (38.56)	95.25 (38.23)	88.79 (25.64)	0.47	0.92	95.83 (35.85)	93.79 (40.79)	91.53 (29.58)	0.75	0.86
**Blood Pressure**										
SBP (mmHg)	117.49 (18.8)	117.14 (17.53)	114.56 (14.7)	0.11	0.05	116.55 (20.02)	117.17 (13.72)	116.09 (16.86)	0.94	0.98
DBP (mmHg)	76.17 (14.19)	77.87(11.18)	77.31 (10.00)	0.68	0.79	75.69 (15.04)	78.07 (9.39)	77.83 (10.20)	0.43	0.86
**Qualitative variable**										
**Sex**										
male	23 (22.7)	41 (40.5)	37 (36.8)	0.03	0.99	26 (25.7)	41 (40.5)	34 (33.8)	0.33	0.99
female	60 (40.3)	36 (24.2)	53 (35.6)			61 (40.9)	48 (32.2)	40 (26.8)		
**PA (Mets)**										
Low	75 (38.7)	56 (28.9)	63 (32.5)	0.96	0.67	74 (38.1)	69 (35.6)	51 (26.3)	0.44	0.68
Moderate	8 (14.2)	21 (37.5)	27 (48.3)			13 (23.2)	20 (35.7)	23 (41.1)		
**Marriage status**										
Single	20 (24.2)	27 (32.5)	36 (43.3)	0.55	0.51	26 (31.3)	28 (33.7)	29 (35)	0.53	0.62
Married	63 (37.7)	50 (29.9)	54 (32.3)			61 (36.5)	61 (36.5)	45 (26.9)		
**Education level**										
≤ Diploma	26 (40)	17 (26.2)	22 (33.8)	0.06	0.05	18 (27.7)	31 (47.7)	16 (24.6)	0.1	0.13
University education	57 (30.8)	60 (32.4)	68 (36.8)			69 (37.4)	58 (31.3)	58 (31.3)		
**Job group**										
Office worker	40 (33.6)	37 (31.1)	42 (35.3)	0.46	0.51	44 (37)	41 (34.5)	34 (28.6)	0.99	0.92
Health service	25 (45.5)	16 (29.1)	14 (25.5)			23 (41.8)	21 (38.2)	11 (20)		
Technical worker	8 (25.8)	11 (35.4)	12 (38.8)			6 (20)	12 (40)	12 (40)		
Guarding worker	10 (22.4)	13 (28.8)	22 (48.8)			14 (30)	15 (32)	17 (38)		
**Socio-economic status**										
Low	4 (13.3)	12 (40)	14 (46.7)	0.29	0.31	3 (10)	14 (46)	13 (44)	0.69	0.61
Moderate	67 (37.2)	55 (30.6)	58 (32.2)			69 (38.3)	62 (34.4)	49 (27.2)		
High	12 (30)	10 (25)	18 (45)			15 (34.8)	16 (37.2)	12 (28)		
**Smoking**										
Yes	31 (38.3)	21 (25.9)	29 (35.8)	0.63	0.66	31 (38.3)	31 (38.3)	19 (23.5)	0.59	0.61
No	52 (30.7)	56 (33.1)	61 (36.2)			56 (33. 2)	58 (34.3)	55 (32.5)		

P-values^*^ is found by ANCOVA and adjusted for age, sex, BMI, physical activity, and total energy intake. PA, physical activity; BMI, body mass index; HC, hip circumference; WC, waist circumference; WHR, weight to hip ratio; FBS, fasting blood sugar; TC, total cholesterol; TG, triglyceride; HDL, high-density lipoprotein; LDL, low-density lipoprotein. Quantitative variables as means (± SD) obtained from the independent t-test. Qualitative variables N (%) obtained from the chi-square analysis. P < 0.05 were considered significant. Bold values indicate *p* < 0.05.

We observed a significant difference between the tertiles of nitrite intake and LDL levels (*P* = 0.03), so with higher intakes of nitrite, mean LDL levels decreased. We did not observe any significant differences with other variables among the tertiles of nitrate and nitrite.

### 3.4 Dietary intake of the participants among the levels of TMAO and KYN tertiles

The dietary intake of the study population among the tertiles of TMAO and KYN is listed in Table 3. In our study, the average energy intake of people who were in the third tertile of TMAO and KYN was higher than that of people who were in the first tertile (*P* < 0.05).

**Table 3:** Characteristics of participants among tertiles of dietary nitrate and nitrite intake.

**Variables**	**TMAO (pg/ml)**					**KYN(nmol/l)**				
	**T1 (** ≤ **23.77)** ***n** =* **80**	**T2 (23.77-51.02)** ***n** =* **78**	**T3 (**≥**51.02)** ***n** =* **92**	**P value**	**P value** ^*^	**T1 (** ≤ **225.35)** ***n** =* **86**	**T2 (225.35-417.41)** ***n** =* **75**	**T3 (**≥**417.41)** ***N** =* **89**	**P value**	**P value** ^*^
Energy (kcal/day)	2533.72 (736.63)	2599.24 (623.15)	2799.74 (702.12)	**0.02**	**-**	2530.20 (679.68)	2668.01 (628.26)	2729.16(783.19)	**0.04**	**-**
**Macronutrient**										
Cho (gr/day)	358.59(106.42)	368.75(104.94)	395.85(99.67)	0.09	**< 0.001**	361.21 (109.03)	378.39 (92.25)	382.65 (112.92)	0.46	**< 0.001**
Fat (gr/day)	82.96 (31.1)	84.49 (23.16)	91.03 (32.02)	0.23	**< 0.001**	81.28 (24.93)	86.73 (28.08)	90.37 (34.15)	0.21	**< 0.001**
Protein (gr/day)	99.02 (25.95)	96.62 (28.63)	108.12(30.88)	**0.04**	**< 0.001**	96.27 (25.98)	102.05 (28.18)	105.29 (32.16)	0.2	**< 0.001**
**Minerals**										
Ca (mg/day)	1045.97 (391.04)	919.42 (320.96)	964.81 (390.87)	0.12	**< 0.001**	936.3 (343.9)	1021.9(412.97)	975.41(351.58)	0.4	**< 0.001**
P (mg/day)	1146.51 (378.66)	1131.04 (381.29)	1268.74 (473.68)	0.1	**< 0.001**	1138.16(373.2)	1234.03(442.43)	1175.7 (430.23)	0.42	**< 0.001**
Mg (mg/day)	248.17 (83.6)	231.71 (71.97)	235.04 (77.76)	0.42	**< 0.001**	236.41 (80.71)	248.02 (77.32)	230.71(74.91)	0.42	**< 0.001**
Fe (mg/day)	19.23 (6.23)	19.78 (5.89)	20.81 (5.96)	0.29	**< 0.001**	20.31 (5.53)	20.65 (6.53)	18.78 (6.03)	0.17	**< 0.001**
Na (mg/day)	3274.37 (978.86)	3005.78 (1037.13)	3140.41 (868.03)	0.26	**< 0.001**	3146.63(942.3)	3230.34(1087.57)	3034.34(878.44)	0.52	**< 0.001**
K (mg/day)	3147.59 (1124.34)	2936.66 (1040.15)	2933.67 (1124.89)	0.43	**< 0.001**	2957.02(1146.76)	3197.87(1104.47)	2873.5(1010.05)	0.21	**< 0.001**
Zinc (mg/day)	8.21 (2.8)	7.38 (2.33)	7.61 (2.29)	0.13	**< 0.001**	7.71 (2.58)	7.98 (2.6)	7.51 (2.32)	0.57	**< 0.001**
Copper (mg/day)	1.24 (0.51)	1.17 (0.53)	1.17 (0.46)	0.64	**< 0.001**	1.24 (0.51)	1.21 (0.49)	1.12 (0.49)	0.33	**< 0.001**
**Vitamins**										
A (IU/day)	1851.57 (1600.67)	1868.07 (1378.57)	2023.63 (2380.41)	0.83	0.22	1792.42(1617.24)	2149.16(1704.72)	1804 (2073.79)	0.43	**0.02**
D (μg/day)	1.63 (1.5)	1.4 (1.24)	1.59 (1.4)	0.57	**0.01**	1.42 (1.17)	1.66 (1.66)	1.55 (1.29)	0.11	**0.01**
E (mg/day)	3.14 (1.02)	34.08 (1.05)	3.17(1.15)	0.87	0.18	3.07(1.02)	3.21 (1.1)	3.11(1.1)	0.36	**< 0.001**
K (mg/day)	166.19 (83.53)	147.99 (63.06)	142.84(58.91)	0.13	**< 0.001**	149.06 (1.17)	161.17 (76.12)	148.02 (60.4)	0.29	**< 0.001**
B1 (mg/day)	2.26 (0.56)	2.05 (0.61)	2.16 (0.6)	0.1	**< 0.001**	2.16 (0.53)	2.2 (0.67)	2.09 (0.6)	0.18	**< 0.001**
B2 (mg/day)	1.83 (0.71)	1.62 (0.61)	1.68 (0.68)	0.16	**< 0.001**	1.67 (0.6)	1.8 (0.72)	1.67 (0.63)	0.47	**< 0.001**
B3 (mg/day)	29.30 (7.95)	26.41 (8.29)	26.99 (6.97)	0.07	**< 0.001**	28.25 (7.16)	28.32 (9.07)	26.03 (7.2)	0.16	**< 0.001**
B6 (mg/day)	1.34 (0.57)	1.34 (0.54)	1.5 (0.73)	0.23	**< 0.001**	1.4 (0.56)	1.48(0.71)	1.3 (0.58)	0.27	**< 0.001**
B12 (μg/day)	3.34 (2.07)	3.48 (2.51)	4 (2.6)	0.26	**< 0.001**	3.39 (2.22)	4.11 (2.83)	3.37 (2.15)	0.14	**< 0.001**
B9 (μg/day)	305.95(110.13)	281.93 (97.87)	284.63(107.13)	0.34	**< 0.001**	293.67(116.24)	299.83 (98.2)	278.74 (98.33)	0.5	**< 0.001**
**Food groups**										
Grain	558.80(171.47)	503.45(176.27)	540.52(138.76)	0.12	**< 0.001**	538.61(153.51)	532.18 (18.83)	528.77 (154.12)	0.23	**< 0.001**
Fruits	308.48 (151.8)	294.92(183.82)	291.05(203.94)	0.14	**< 0.001**	299.05 (177.35)	1319.87 (195.45)	275.98 (165.06)	0.18	**< 0.001**
Vegetables	349.54(233.91)	347.15(185.32)	339.71(189.05)	0.15	**< 0.001**	363.31 (173.04)	343.73 (226.93)	330.65 (202.68)	0.24	**< 0.001**
Meats	44.31 (19.78)	48.43 (30.11)	50.96 (29.38)	0.37	**< 0.001**	42.84 (20.6)	49.78 (28.6)	51.06 (30.34)	0.17	**< 0.001**
Dairy	366.57 (250.3)	316.65(186.87)	327.26 (212.1)	0.16	**< 0.001**	317.54 (211.4)	364.34 (248.88)	331.1 (189.97)	0.32	**< 0.001**

All data are presented as mean (±SD). P < 0.05 were considered significant. P-value^*^ for adjustment model, based on energy intake. Bold values indicate *p* < 0.05.

All macronutrients before and after adjusting for energy intake had an increased trend across increasing levels of TMAO and KYN. It was found that after adjusting for energy intake, there were significant differences between groups of TMAO and KYN and micronutrients (*P* < 0.05).

We observed that the mean consumption of vegetables in tertile 3 TMAO compared with tertile 1 reduced (*P* < 0.05), but in the case of meat consumption, this difference was increased so that in the third level of TMAO meat consumption was 50.96 (29.38) mg/day compared to the first level of 44.31 (19.78) mg/day. Moreover, this study indicates a significant difference between the consumption of food groups among tertiles of KYN after adjusting for energy intake (*P* < 0.001).

### 3.5 Association between intake of total nitrite and nitrate and animal and plant sources with the TMAO levels

The association between intake of total nitrite and nitrates and animal and plant sources with the levels of TMAO is shown in Table 4.

**Table 4:** Association between intake of total nitrite and nitrates and animal and plant sources with TMAO levels.

**Variables**		**TMAO (pg/ml)**						
		**T2 (23.77-51.02)**			**T3 (**≥**51.02)**			**P trend**
		**OR**	**95% CI**	***P*** **-value**	**OR**	**95% CI**	***P-*** **value**	
**Nitrate total**								
T2	Crude	0.81	0.37, 1.8	0.68	1.03	0.45, 2.34	0.93	0.68
	Adjusted	1.86	0.66, 5.21	0.23	1.6	0.64, 3.98	0.3	
T3	Crude	0.79	0.34, 1.79	0.57	0.89	0.38, 2.1	0.79	0.1
	Adjusted	0.73	0.41, 3.51	0.72	0.75	0.49, 3.23	0.63	
**Animal source**								
T2	Crude	1.02	0.39, 2.04	0.8	2.32	0.99, 5.41	**0.05**	**0.03**
	Adjusted	1.76	0.65, 4.75	0.25	3.38	1.35, 8.48	**< 0.001**	
T3	Crude	1.07	0.31, 1.6	0.4	1.23	0.51, 2.97	**0.06**	**0.04**
	Adjusted	1.01	0.37, 2.69	0.98	1.51	0.59, 3.88	**0.03**	
**Plant source**								
T2	Crude	1.21	0.43, 3.41	0.71	1.06	0.43, 3.41	0.89	0.3
	Adjusted	0.62	0.27, 1.4	0.25	0.77	0.34, 1.76	0.54	
T3	Crude	1.04	0.35, 3.09	0.94	1.32	0.52, 3.35	0.54	0.11
	Adjusted	0.73	0.31, 1.72	0.47	1.02	0.42, 2.35	0.89	
**Nitrite total**								
T2	Crude	0.64	0.27, 1.5	0.31	1.17	0.53, 2.55	0.69	0.67
	Adjusted	1.83	0.53, 6.27	0.33	2.07	0.82, 5.21	0.12	
T3	Crude	0.67	0.28, 1.57	0.35	0.79	0.34, 1.82	0.59	0.77
	Adjusted	1.23	0.36, 4.19	0.73	1.11	0.42, 2.89	0.82	
**Animal source**								
T2	Crude	1.08	0.39, 1.99	0.76	1.75	0.77, 4.01	0.18	**0.02**
	Adjusted	1.7	0.63, 4.56	0.28	2.56	1.03, 6.33	**0.04**	
T3	Crude	1.17	0.38, 2.19	0.76	1.27	0.53, 3.01	0.08	**0.02**
	Adjusted	1.36	0.5, 3.66	0.54	1.73	0.67, 4.43	**0.02**	
**Plant source**								
T2	Crude	1.76	0.56, 5.49	0.32	1.9	0.74, 4.87	0.17	0.65
	Adjusted	0.7	0.31, 1.6	0.4	1.12	0.49, 2.54	0.77	
T3	Crude	1.24	0.39, 3.99	0.7	1.24	0.46, 3.27	0.66	0.87
	Adjusted	0.73	0.31, 1.69	0.46	0.86	0.36, 2.04	0.73	

P < 0.05 were considered significant. P-value^*^ for adjustment model, based on energy intake and physical activity, BMI, sex and age. Tertile 1 of level of TMAO (≤ 23.77 pg/ml) and Tertile 1 of total nitrate (≤ 540.58 mg), Animal Nitrate (≤ 19.28 mg), Plant Nitrate (≤ 516.95 mg), total nitrite (≤ 10.62 mg), Animal Nitrite (≤ 5.28 mg), Plant Nitrite (≤ 5.02 mg) were as reference group. Cut points for Tertile 2 of total Nitrate (540.58, 750.23 mg), Animal source (19.24, 26.98 mg), Plant source (516.95, 726.32 mg), Tertile 3 of total Nitrate (≥ 750.23), Animal source (≥ 26.98 mg), Plant source (≥726.32 mg). Tertile 2 of total Nitrite (10.62, 13.87 mg), Animal source (5.28, 7.50 mg), Plant source (5.02, 6.50 mg), Tertile 3 of total Nitrite (≥ 13.87), Animal source (≥ 7.50 mg), Plant source (≥ 6.50 mg). Animal source: Dairy + Meat + Poultry + Fish + Processed Meat. Plant Source: Grains + legumes + nuts + fruits + leafy vegetables + root vegetables+ Starchy vegetables + other vegetables. Bold values indicate *p* < 0.05.

In the crude model, our results showed that people who were in the second tertile adherence to receiving nitrate from animal sources in comparison to the first tertile had higher odds of having the highest level of TMAO (≥51.02 pg/ml) (OR = 2.32, 95% CI = 0.99,5.41, *P* = 0.05), and when we added the potential confounders to the model, the odds of the highest levels of TMAO 3.38 times increased, which was significant (OR = 3.38, 95% CI = 1.35,8.48, *P* < 0.001, P for trend = 0.03).

Subjects with the highest nitrate intake from animal sources had odds 1.23 times higher level of having the highest level of TMAO compared to the reference group, which was marginally significant. However, after adjusting for confounders, this association increased statistically significantly (OR = 1.51, 95% CI = 0.59–3.88, *P* = 0.03, P for trend = 0.04).

Our results showed that after adjustment, subjects who were in the second and third tertile of nitrite intake from animal sources had the lowest intake odds of the highest level of TMAO (≥51.02 pg/ml) 2.56 and 1.73 times increased, respectively, and that there was a significant difference (P for trend < 0.05).

No significant association was seen between the intake total and plant source of nitrite and the levels of TMAO (*P* > 0.05).

### 3.6 Association between intake of total nitrite and nitrate and animal and plant sources with the KYN levels

The association between intake of total nitrite and nitrate and animal and plant sources among the tertile levels of KYN is shown in Table 5.

**Table 5:** Association between intake of total Nitrite and Nitrate and animal and plant sources with KYN levels.

		**KYN(nmol/l)**						
		**T2 (225.35-417.41)**			**T3 (**≥**417.41)**			
**Variables**		**OR**	**95% CI**	***P*** **-value**	**OR**	**95% CI**	***P-*** **value**	**P trend**
**Nitrate total**								
T2	Crude	1.75	0.79, 3.87	0.16	1.08	0.46, 2.56	0.84	0.21
	Adjusted	2.02	0.72, 5.7	0.81	1.26	0.49, 3.2	0.62	
T3	Crude	0.89	0.35, 1.84	0.61	0.97	0.5, 2.46	0.79	0.69
	Adjusted	1.13	0.39, 3.23	0.81	1.41	0.58, 3.38	0.43	
**Animal source**								
T2	Crude	1.61	0.7, 3.69	0.25	2.21	0.93, 5.22	**0.07**	**0.04**
	Adjusted	1.62	0.61, 4.35	0.33	2.29	0.92, 5.69	**0.06**	
T3	Crude	0.84	0.37, 1.9	0.68	1.47	0.64, 3.34	0.15	**0.06**
	Adjusted	1.13	0.43, 2.97	0.8	1.75	0.73, 4.17	**0.02**	
**Plant source**								
T2	Crude	2.19	0.76, 6.31	0.14	1.39	0.55, 3.53	0.48	0.18
	Adjusted	1.82	0.81, 4.11	0.14	1.17	0.49, 2.76	0.71	
T3	Crude	1.24	0.42, 3.66	0.68	1.77	0.74, 4.23	0.19	0.47
	Adjusted	0.85	0.36, 1.99	0.71	1.38	0.62, 3.07	0.41	
**Nitrite total**								
T2	Crude	1.54	0.66, 3.6	0.31	1.41	0.63, 3.17	0.4	0.19
	Adjusted	1.85	0.55, 6.22	0.31	1.7	0.67, 4.34	0.26	
T3	Crude	0.6	0.25, 1.44	0.25	0.78	0.36, 1.7	0.53	0.79
	Adjusted	0.75	0.22, 2.55	0.65	1.09	0.37, 2.26	0.85	
**Animal source**								
T2	Crude	1.37	0.59, 3.17	0.45	2.3	0.98, 5.43	**0.08**	**0.04**
	Adjusted	1.24	0.4, 3.33	0.66	2.25	0.89, 5.65	**0.05**	
T3	Crude	1.09	0.33, 1.68	0.48	1.3	0.57, 2.97	0.32	**0.05**
	Adjusted	1.16	0.34, 2.38	0.85	1.45	0.6, 3.52	**0.03**	
**Plant source**								
T2	Crude	2.23	0.69, 7.16	0.17	1.55	0.6, 4.03	0.36	0.2
	Adjusted	1.77	0.75, 4.14	0.18	1.22	0.52, 2.85	0.64	
T3	Crude	1.07	0.3, 3.33	0.89	0.76	0.3, 1.88	0.55	0.67
	Adjusted	0.76	0.33, 1.75	0.52	0.59	0.26, 1.33	0.2	

P < 0.05 were considered significant. P-value^*^ for adjustment model, based on energy intake and physical activity, BMI, sex and age. Tertile 1 of level of TMAO (≤ 23.77 pg/ml) and Tertile 1 of total nitrate (≤ 540.58 mg), Animal Nitrate (≤ 19.28 mg), Plant Nitrate (≤ 516.95 mg), total nitrite (10.62 ≤ mg), Animal Nitrite (≤ 5.28 mg), Plant Nitrite (≤ 5.02 mg) were as reference group. Cut points for Tertile 2 of total Nitrate (540.58, 750.23 mg), Animal source (19.24, 26.98 mg), Plant source (516.95, 726.32 mg), Tertile 3 of total Nitrate (≥ 750.23), Animal source (≥ 26.98 mg), Plant source (≥726.32 mg). Tertile 2 of total Nitrite (10.62, 13.87 mg), Animal source (5.28, 7.50 mg), Plant source (5.02, 6.50 mg), Tertile 3 of total Nitrite (≥ 13.87), Animal source (≥ 7.50 mg), Plant source (≥6.50 mg). Animal source: Dairy + Meat + Poultry + Fish + Processed Meat. Plant Source: Grains + legumes + nuts + fruits + leafy vegetables + root vegetables+ Starchy vegetables + other vegetables. Bold values indicate *p* < 0.05.

No significant association was seen between the intake total and plant source of nitrite and nitrate with KYN levels before and after adjustment (*P* > 0.05).

Subjects who were in the second tertile of animal sources of nitrate had a 2.21-fold increase in nitrate levels in comparison with the reference group (OR = 2.21, 95% CI = 0.93, 5.22, *P* = 0.07) that was marginally significant; after adjusting for confounders, this difference was significant (OR = 2.29, 95% CI = 0.92, 5.69, P for trend = 0.04). After adjusting for confounders, subjects in the third tertile of animal sources of nitrate only had odds of having the highest level of KYN (≥417.41 nmol/L), which was 1.75 times higher in comparison with the reference group.

Our results showed that, after adjustment, subjects in the second (5.28–7.50 mg/day) and third tertile (≥7.50 mg/day) of nitrite intake from animal sources had the lowest intake of having the highest level of KYN 2.25 and 1.45 times increased, respectively, indicating a significant difference.

### 3.7 Association between intake of total nitrite and nitrate and animal and plant sources with the TMAO levels in men

The association between intake of total nitrite and nitrates and animal and plant sources with the levels of TMAO in men is shown in Table 6.

**Table 6:** Association between intake of total nitrite and nitrates and animal and plant sources with TMAO levels in men.

**Variables**		**TMAO (pg/ml)**						
		**T2 (23.77-51.02)**			**T3 (**≥**51.02)**			**P trend**
		**OR**	**95% CI**	***P*** **-value**	**OR**	**95% CI**	***P*** **-value**	
**Nitrate total**								
T2	Crude	1.07	0.39, 1.95	0.64	1.05	0.43, 2.03	0.33	0.13
	Adjusted	1.26	0.48, 2.25	0.44	1.27	0.71, 2.56	0.10	
T3	Crude	0.79	0.36, 1.89	0.35	0.85	0.33, 3.20	0.52	0.39
	Adjusted	1.09	0.55, 2.30	0.36	0.74	0.58, 3.28	0.80	
**Animal source**								
T2	Crude	3.66	1.77, 5.26	0.10	2.00	1.27, 4.88	0.12	**0.04**
	Adjusted	2.26	1.66, 4.52	**0.03**	1.06	1.11, 3.44	**0.05**	
T3	Crude	2.66	1.13, 3.30	0.11	1.66	1.29, 4.44	0.06	**0.03**
	Adjusted	2.25	1.33, 3.96	**0.01**	1.76	1.59, 4.26	**0.03**	
**Plant source**								
T2	Crude	1.04	0.23, 4.70	0.93	1.88	0.88, 4.21	0.22	0.08
	Adjusted	0.53	0.10, 2.95	0.47	1.78	0.76, 4.26	0.41	
T3	Crude	0.75	0.49, 3.11	0.53	1.14	0.53, 3.55	0.55	0.47
	Adjusted	0.89	0.40, 2.26	0.08	0.98	0.49, 2.26	0.77	
**Nitrite total**								
T2	Crude	0.88	0.41, 8.61	0.41	1.21	0.55, 2.89	0.66	0.07
	Adjusted	1.89	0.55, 6.66	0.07	2.18	0.88, 4.21	0.50	
T3	Crude	0.79	0.38, 1.69	0.55	0.78	0.35, 2.78	0.28	0.32
	Adjusted	1.33	0.46, 3.21	0.08	1.12	0.49, 2.99	0.57	
**Animal source**								
T2	Crude	1.09	0.49, 2.01	0.45	1.85	0.88, 3.89	0.10	**0.01**
	Adjusted	1.80	0.69, 3.59	0.32	2.42	1.22, 5.36	**0.02**	
T3	Crude	1.37	0.48, 2.69	0.26	1.18	1.08, 2.02	**0.05**	**0.01**
	Adjusted	1.46	0.52, 3.52	0.22	1.33	1.09, 3.26	**0.02**	
**Plant source**								
T2	Crude	1.79	0.55, 4.42	0.22	1.99	0.79, 4.89	0.17	0.14
	Adjusted	0.85	033, 1.64	0.11	1.12	0.52, 2.65	0.12	
T3	Crude	1.25	0.38, 3.29	0.17	1.25	0.59, 3.89	0.23	0.11
	Adjusted	0.63	0.38, 2.62	0.12	0.79	0.46, 2.44	0.33	

P < 0.05 were considered significant. P-value for adjustment model, based on energy intake and physical activity, BMI, sex and age. Tertile 1 of level of TMAO (≤ 23.77 pg/ml) and Tertile 1 of total nitrate (≤ 540.58 mg), Animal Nitrate (≤ 19.28 mg), Plant Nitrate (≤ 516.95 mg), total nitrite (≤ 10.62 mg), Animal Nitrite (≤ 5.28 mg), Plant Nitrite (≤ 5.02 mg) were as reference group. Cut points for Tertile 2 of total Nitrate (540.58, 750.23 mg), Animal source (19.24, 26.98 mg), Plant source (516.95, 726.32 mg), Tertile 3 of total Nitrate (≥ 750.23), Animal source (≥ 26.98 mg), Plant source (≥726.32 mg). Tertile 2 of total Nitrite (10.62, 13.87 mg), Animal source (5.28, 7.50 mg), Plant source (5.02, 6.50 mg), Tertile 3 of total Nitrite (≥ 13.87), Animal source (≥ 7.50 mg), Plant source (≥ 6.50 mg). Animal source: Dairy + Meat + Poultry + Fish + Processed Meat. Plant Source: Grains + legumes + nuts + fruits + leafy vegetables + root vegetables+ Starchy vegetables + other vegetables. Bold values indicate *p* < 0.05.

### 3.8 Association between intake of total nitrite and nitrate and animal and plant sources with the TMAO levels in women

The association between intake of total nitrite and nitrates and animal and plant sources with the levels of TMAO in women is shown in Table 7.

**Table 7:** Association between intake of total nitrite and nitrates and animal and plant sources with TMAO levels in women.

**Variables**		**TMAO (pg/ml)**						
		**T2 (23.77-51.02)**			**T3 (**≥**51.02)**			**P trend**
	**OR**	**95% CI**	***P*** **-value**	**OR**	**95% CI**	***P*** **-value**	
**Nitrate total**								
T2	Crude	0.46	0.23, 1.76	0.39	0.64	0.28, 1.80	0.40	0.92
	Adjusted	0.82	0.27, 2.52	0.73	0.70	0.22, 2.14	0.76	
T3	Crude	0.58	0.23, 1.47	0.25	0.72	0.22, 1.81	0.48	0.54
	Adjusted	0.95	0.27, 3.28	0.94	0.82	0.24, 2.83	0.53	
**Animal source**								
T2	Crude	2.22	0.83, 5.92	0.11	1.66	0.61, 4.51	**0.05**	**0.03**
	Adjusted	1.95	0.68, 5.57	0.21	1.66	0.58, 4.76	**0.04**	
T3	Crude	1.33	0.50, 3.49	0.55	1.46	0.54, 3.60	0.07	**0.04**
	Adjusted	1.77	0.73, 2.53	0.67	1.89	0.88, 3.74	**0.04**	
**Plant source**								
T2	Crude	1.21	0.45, 3.30	0.69	1.25	0.47, 3.27	0.38	0.87
	Adjusted	0.95	0.32, 2.87	0.45	1.15	0.39, 3.37	0.79	
T3	Crude	2.22	0.86, 5.71	0.09	1.52	0.58, 3.97	0.65	0.67
	Adjusted	1.61	0.46, 5.57	094	1.42	0.41, 4.47	0.57	
**Nitrite total**								
T2	Crude	0.98	0.38, 2.47	0.96	0.80	0.33, 2.33	0.65	0.87
	Adjusted	1.52	0.50, 4.55	0.77	0.99	0.33, 2.96	0.89	
T3	Crude	0.60	0.22, 1.62	0.31	0.85	0.30, 2.09	0.75	0.49
	Adjusted	1.22	0.30, 4.90	0.45	1.09	0.28, 4.15	0.99	
**Animal source**								
T2	Crude	1.28	0.48, 3.39	0.61	1.21	0.45, 3.21	0.17	0.21
	Adjusted	1.06	0.37, 3.00	0.90	1.17	0.42, 3.25	**0.05**	
T3	Crude	1.11	0.29, 2.86	0.87	1.12	0.32, 2.83	0.11	**0.03**
	Adjusted	1.18	0.21, 2.19	0.52	1.25	0.43, 2.91	**0.02**	
**Plant source**								
T2	Crude	1.73	0.64, 4.62	0.15	1.33	0.49, 3.57	0.57	0.95
	Adjusted	1.45	0.48, 4.39	0.50	1.34	0.44, 4.09	0.59	
T3	Crude	1.52	0.77, 5.18	0.28	1.07	0.25, 4.29	0.28	0.80
	Adjusted	1.21	0.30, 4.76	0.78	1.14	0.42, 6.44	0.47	

P < 0.05 were considered significant. P-value for adjustment model, based on energy intake and physical activity, BMI, sex and age. Tertile 1 of level of TMAO (≤ 23.77 pg/ml) and Tertile 1 of total nitrate (≤ 540.58 mg), Animal Nitrate (≤ 19.28 mg), Plant Nitrate (≤ 516.95 mg), total nitrite (≤ 10.62 mg), Animal Nitrite (≤ 5.28 mg), Plant Nitrite (≤ 5.02 mg) were as reference group. Cut points for Tertile 2 of total Nitrate (540.58, 750.23 mg), Animal source (19.24, 26.98 mg), Plant source (516.95, 726.32 mg), Tertile 3 of total Nitrate (≥ 750.23), Animal source (≥ 26.98 mg), Plant source (≥726.32 mg). Tertile 2 of total Nitrite (10.62, 13.87 mg), Animal source (5.28, 7.50 mg), Plant source (5.02, 6.50 mg), Tertile 3 of total Nitrite (≥ 13.87), Animal source (≥ 7.50 mg), Plant source (≥ 6.50 mg). Animal source: Dairy + Meat + Poultry + Fish + Processed Meat. Plant Source: Grains + legumes + nuts + fruits + leafy vegetables + root vegetables+ Starchy vegetables + other vegetables. Bold values indicate *p* < 0.05.

After adjusting for confounders, men and women who were in the second and third tertiles of animal sources of nitrate and nitrite had the highest level of TMAO (≥51.02) compared to the reference group (*P* < 0.05).

### 3.9 Association between intake of total nitrite and nitrate and animal and plant sources with the kynurenine levels in men

The association between intake of total nitrite and nitrates and animal and plant sources with the levels of kynurenine in men is shown in Table 8.

**Table 8:** Association between intake of total nitrite and nitrates and animal and plant sources with KYN levels in men.

**Variables**		**KYN (pg/ml)**						
		**T2 (225.35-417.41)**			**T3 (**≥**417.41)**			**P trend**
	**OR**	**95% CI**	***P*** **-value**	**OR**	**95% CI**	***P*** **-value**	
**Nitrate total**								
T2	Crude	1.85	0.78, 3.81	0.78	1.08	0.56, 2.63	0.93	0.75
	Adjusted	2.23	0.75, 4.15	0.72	1.36	0.45, 3.29	0.41	
T3	Crude	0.96	0.49, 1.79	0.51	0.91	0.35, 2.49	0.51	0.75
	Adjusted	1.26	0.35, 3.29	0.57	1.24	0.68, 3.18	0.73	
**Animal source**								
T2	Crude	1.69	0.78, 3.26	0.16	2.28	1.02, 4.22	**0.04**	**0.04**
	Adjusted	1.85	0.79, 4.25	0.18	2.26	1.12, 5.32	**0.03**	
T3	Crude	1.15	0.44, 2.95	0.10	2.47	1.29, 4.92	**0.04**	**0.03**
	Adjusted	1.33	0.65, 2.25	**0.02**	2.75	1.48, 5.42	**0.01**	
**Plant source**								
T2	Crude	2.29	0.86, 5.32	0.42	1.38	0.59, 3.22	0.84	0.92
	Adjusted	1.82	0.88, 4.15	0.79	1.12	0.45, 2.95	0.77	
T3	Crude	1.34	0.45, 3.27	0.73	1.75	0.74, 4.21	0.63	0.69
	Adjusted	1.20	0.44, 3.16	0.47	1.26	0.56, 3.17	0.63	
**Nitrite total**								
T2	Crude	1.55	0.66, 3.62	**0.03**	1.44	0.66, 3.19	0.35	0.88
	Adjusted	1.75	0.45, 4.99	**0.02**	1.78	0.68, 4.26	0.56	
T3	Crude	1.12	0.43, 3.97	0.21	0.89	0.49, 2.25	0.30	0.28
	Adjusted	1.10	0.48, 3.26	0.23	0.95	0.55, 2.49	0.28	
**Animal source**								
T2	Crude	1.39	0.69, 3.19	0.08	2.39	1.02, 5.45	0.08	**0.03**
	Adjusted	1.29	0.49, 3.69	0.28	2.55	1.01, 5.12	**0.05**	
T3	Crude	1.10	0.38.1.96	0.25	1.35	0.69, 2.95	**0.05**	**0.03**
	Adjusted	1.26	0.45, 2.98	0.58	1.59	0.72, 2.56	**0.03**	
**Plant source**								
T2	Crude	2.33	0.68, 4.15	0.17	1.45	0.62, 4.32	0.93	0.51
	Adjusted	1.78	0.79, 4.10	0.12	1.32	0.55, 2.95	0.80	
T3	Crude	1.08	0.33, 2.32	0.99	0.78	0.38, 1.98	0.99	0.65
	Adjusted	0.77	0.39, 2.79	0.83	0.69	0.29, 1.43	0.75	

P < 0.05 were considered significant. P-value for adjustment model, based on energy intake and physical activity, BMI, sex and age. Tertile 1 of level of TMAO (≤ 23.77 pg/ml) and Tertile 1 of total nitrate (≤ 540.58 mg), Animal Nitrate (≤ 19.28 mg), Plant Nitrate (≤ 516.95 mg), total nitrite (10.62 ≤ mg), Animal Nitrite (≤ 5.28 mg), Plant Nitrite (≤ 5.02 mg) were as reference group. Cut points for Tertile 2 of total Nitrate (540.58, 750.23 mg), Animal source (19.24, 26.98 mg), Plant source (516.95, 726.32 mg), Tertile 3 of total Nitrate (≥ 750.23), Animal source (≥ 26.98 mg), Plant source (≥726.32 mg). Tertile 2 of total Nitrite (10.62, 13.87 mg), Animal source (5.28, 7.50 mg), Plant source (5.02, 6.50 mg), Tertile 3 of total Nitrite (≥ 13.87), Animal source (≥ 7.50 mg), Plant source (≥6.50 mg). Animal source: Dairy + Meat + Poultry + Fish + Processed Meat. Plant Source: Grains + legumes + nuts + fruits + leafy vegetables + root vegetables+ Starchy vegetables + other vegetables. Bold values indicate *p* < 0.05.

### 3.10 Association between intake of total nitrite and nitrate and animal and plant sources with the kynurenine levels in women

The association between intake of total nitrite and nitrates and animal and plant sources with the levels of kynurenine in women is shown in Table 9.

**Table 9:** Association between intake of total nitrite and nitrates and animal and plant sources with KYN levels in women.

		**KYN (pg/ml)**						
**Variables**		**T2 (225.35-417.41)**			**T3 (**≥**417.41)**			**P trend**
	**OR**	**95% CI**	***P-*** **value**	**OR**	**95% CI**	***P-*** **value**	
**Nitrate total**								
T2	Crude	1.13	0.40, 3.19	0.81	1.03	0.28, 1.86	0.94	0.94
	Adjusted	1.32	0.39, 3.80	0.71	1.21	0.41, 3.58	0.72	
T3	Crude	1.67	0.68, 4.09	0.26	0.07	0.38, 2.77	0.51	0.3
	Adjusted	2.11	0.62, 7.21	0.23	1.04	0.29, 3.54	0.95	
**Animal source**								
T2	Crude	1.02	0.32, 2.68	0.25	1.78	0.65, 4.83	**0.02**	**0.02**
	Adjusted	1.1	0.40, 3.06	0.34	1.65	0.57, 4.74	**0.03**	
T3	Crude	0.75	0.29, 1.91	0.24	1.3	0.49, 3.46	**0.05**	**0.04**
	Adjusted	1.18	0.46, 2.51	0.12	1.98	0.83, 3.14	**0.04**	
**Plant source**								
T2	Crude	0.69	0.23, 1.47	0.46	1.81	0.65, 4.99	0.25	0.45
	Adjusted	0.58	0.19, 1.78	0.34	1.39	0.45, 4.31	0.56	
T3	Crude	0.59	0.25, 1.87	0.26	1.31	0.50, 3.43	0.58	0.32
	Adjusted	0.46	0.13, 1.57	0.21	0.88	0.54, 3.12	0.84	
**Nitrite total**								
T2	Crude	1.49	0.35, 2.44	0.8	0.54	0.21, 1.36	0.19	0.73
	Adjusted	0.92	0.51, 2.69	0.88	0.63	0.21, 1.85	0.4	
T3	Crude	0.88	0.46, 3.98	0.8	0.72	0.36, 2.01	0.53	0.23
	Adjusted	1.47	0.38, 5.65	0.57	0.94	0.53, 3.76	0.93	
**Animal source**								
T2	Crude	1.19	0.41, 2.72	0.71	1.26	0.54, 3.70	**0.05**	**0.04**
	Adjusted	1.31	0.48, 3.57	0.59	1.13	0.40, 3.22	**0.04**	
T3	Crude	1.05	0.46, 3.08	0.91	1.42	0.47, 3.39	0.1	**0.05**
	Adjusted	1.4	0.45, 4.36	0.55	1.23	0.39, 3.89	**0.03**	
**Plant source**								
T2	Crude	0.51	0.17, 1.18	0.1	0.71	0.25, 2.00	0.52	0.33
	Adjusted	0.37	0.12, 1.12	0.15	0.6	0.18, 1.94	0.92	
T3	Crude	0.45	0.19, 1.34	0.1	1.38	0.52, 3.63	0.5	0.27
	Adjusted	0.49	0.21, 1.28	0.14	0.93	0.23, 3.80	0.39	

P < 0.05 were considered significant. P-value for adjustment model, based on energy intake and physical activity, BMI, sex and age. Tertile 1 of level of TMAO (≤ 23.77 pg/ml) and Tertile 1 of total nitrate (≤ 540.58 mg), Animal Nitrate (≤ 19.28 mg), Plant Nitrate (≤ 516.95 mg), total nitrite (10.62 ≤ mg), Animal Nitrite (≤ 5.28 mg), Plant Nitrite (≤ 5.02 mg) were as reference group. Cut points for Tertile 2 of total Nitrate (540.58, 750.23 mg), Animal source (19.24, 26.98 mg), Plant source (516.95, 726.32 mg), Tertile 3 of total Nitrate (≥ 750.23), Animal source (≥ 26.98 mg), Plant source (≥726.32 mg). Tertile 2 of total Nitrite (10.62, 13.87 mg), Animal source (5.28, 7.50 mg), Plant source (5.02, 6.50 mg), Tertile 3 of total Nitrite (≥ 13.87), Animal source (≥ 7.50 mg), Plant source (≥6.50 mg). Animal source: Dairy + Meat + Poultry + Fish + Processed Meat. Plant Source: Grains + legumes + nuts + fruits + leafy vegetables + root vegetables+ Starchy vegetables + other vegetables. Bold values indicate *p* < 0.05.

Men and women who were in the second and third tertiles of animal sources of nitrate and nitrite after adjusting for confounders had the highest level of KYN (≥417.41) compared to the reference group (*P* < 0.05).

## 4 Discussion

To the best of our knowledge, this cross-sectional study investigated the relationship between dietary nitrates/nitrites intake from animal and plant sources separately with the gut microbial metabolites, including TMAO and KYN. In the findings of our study, it was observed that a high level of nitrate intake from animal sources (≥26.98 mg) can increase the odds of high levels of TMAO (≥51.02 pg/ml) among participants. Furthermore, the findings proved that the highest nitrite intake from animal sources (≥7.50 mg) increases the odds of raising TMAO levels (≥51.02 pg/ml). On the other hand, these results regarding KYN also showed that people with the highest tertile of nitrate and nitrite intake from animal foods have higher odds of increasing KYN (≥417.41 nmol/L).

In line with our study, Meanwhile, Meng Wang et al. in a large, community-based cohort investigated that more meat consumption can augment the risk of atherosclerotic cardiovascular disease via high metalloids such as TMAO (43). In another investigation, the relationship between the consumption of animal foods and TMAO was measured in 271 participants aged over 18 years. The results showed that the consumption of meat, fish, or eggs was not related to the concentration of TMAO, choline, or betaine. However, with increasing consumption of milk and other dairy foods, the plasma TMAO concentration increased (44). In addition, Boutagy et al. (45) investigated the relationship between probiotic supplements and TMAO production following a 4-week high-fat diet in 19 healthy, non-obese men (18–30 years old). The results showed a high-fat diet led to an increase in plasma TMAO. However, probiotic supplementation did not appear to affect plasma TMAO concentrations following a high-fat diet. Furthermore, a study with similar results to our article about KYN was conducted by Karlsson et al. (46) this study investigated the relationship between fish consumption and n-3 long-chain unsaturated fatty acids with plasma metabolites related to the KYN pathway in 2,324 patients with coronary artery disease. The results showed that patients with higher consumption of total fish are more likely to have increased plasma Trp and KYN. Another study with results similar to our study and conducted on elderly men showed a diet that included 2 times the RDA of protein intake for 10 weeks could increase fasting circulating TMAO up to 5-fold in the majority of participants (47). In addition, an observational study showed a positive relationship between circulating TMAO and participants' self-reported dietary intake of fish and red meat (48) and, comparatively, the increase of TMAO in the plasma and urine of omnivores is more than herbivores (19, 49).

However, it seems that the mechanism is not overall obvious and there is a necessity to plan studies for subsequent investigations. Many “non-infectious” diseases such as diabetes, obesity, and cardiovascular disease (CVD) are linked to changes in microbial populations and the microbiome plays a role in a wide variety of diseases (50). New studies have shown that the metabolites obtained from the gut microbiome, including TMAO and KYN, can play a role in the pathogenesis of metabolic syndrome diseases, and the obtained cutoffs can be important in predicting life-threatening metabolic syndrome diseases (51). TMAO is one of the intestinal microbial metabolites, which has many beneficial effects on the heart and blood vessels and human health in general (17). TMAO is a tiny, organic, gut microbiome-generated metabolites, whose blood concentration increments since ingesting phosphatidylcholine and L-carnitine-rich animal foods in the diet like red meat, fish, and eggs (19). There are several possible mechanisms by which dietary nitrate and nitrite may be associated with TMAO levels. In various studies, it has been stated that the dietary increase in red meat leads to a significant increase in the plasma concentration of TMAO, which is a metabolite related to inflammation and CVD (19, 52, 53). Choline, carnitine, phosphatidylcholine, and betaine, which are mostly present in animal food, are metabolized by intestinal microbiota and finally produced to produce trimethylamine (TMA). TMA is absorbed and oxidized by the enzyme FMO3 in the liver and finally forms TMAO (18, 19). Nitrate and nitrite in food can suppress the growth and synthesis of TMA-producing bacteria or compete with TMA precursors and thus reduce TMAO production (20, 21). Nitrate and nitrite are converted into nitric oxide (NO) through enzymatic reactions. NO has antimicrobial properties and can affect the growth and survival, the composition and activity of the intestinal microbiota, and the level of TMAO. Furthermore, NO indirectly affects TMAO levels through its effects on TMA metabolism and is an inhibitor of the FMO3 enzyme, which is responsible for converting TMA into TMAO in the liver (27, 28). By inhibiting FMO3, NO can diminish the production of TMAO (30). TMAO may cause inflammation, and the inflammatory cytokines IL-1 and IL-18 are produced after a significant increase in TMAO and its activation. TMAO also reduces the production of NO and endothelial nitric oxide synthase (eNOS) (54).

In the continuation of the possible mechanisms of the effects of metabolites, the meticulous mechanisms of KYN are unknown. There are various potential routes through which the microbiota could adjust the expression and activity of the KYN pathway. Tryptophan (Trp) is an essential amino acid and is actually the precursor of a number of important physiological metabolites, including melatonin, tryptamine, serotonin, and KYN (55). The most important sources of tryptophan in the diet are fish, chicken, egg, meat, dairy products, milk, bananas, oats, pumpkin, chocolate, sesame seeds, soy, and peanuts (56, 57). KYN is produced through the KYN pathway (KP), which is a series of enzymatic reactions (34).

The KP is responsible for metabolizing ~90% of tryptophan and the rate of conversion depends on the expression of IDO1 (in all tissues) and TDO (only in the liver) (58). It is actually produced ubiquitously from tryptophan under the action of the enzyme (which is mainly hepatic) tryptophan-2,3-dioxygenase (TDO) or indoleamine-2,3-dioxygenase (IDO) (59). The activity of these two enzymes can be influenced by inflammatory molecules, when INF-γ activates the IDO1 results in an increase in the conversion of tryptophan to kynurenine (60). It is possible that the inflammatory process that triggers IDO1 activation and subsequent increase in kynurenine-to-tryptophan ratio may come from a microbial origin (61). Protein synthesis and degradation via the KYN pathway are the primary fates of TRP in the liver (62). It should be noted that hepatocytes are different from other cells in regards to the type of rate-limiting enzyme in the TRP pathway. TRP 2,3-dioxygenase (TDO) is found in the liver, in contrast to indolamine 2,3-dioxygenase 1 or 2 (IDO-1 or IDO-2) which are found in another place. Although IDO and TDO function similarly, their regulation is distinct, leading to distinct physiological roles. TDO's activation mechanism is unique and it is activated by TRP itself, the activation and expression of TDO are almost always continuous, this is the reason why 90% of TRP degradation is caused by it (63). IDO-1 or IDO-2 are the rate-limiting enzymes in cells that are not hepatocytes, although similar in structure, they differ in signaling pathways and expression patterns (64). Dietary nitrate and nitrite can be transformed into nitric oxide, NO regulates IDO (35).

Nevertheless, we could find some relationship between increased consumption of nitrate and nitrite from plant sources with decreasing levels of TMAO and KYN levels, but this relationship was not significant. Vegetables and water are important primary sources of exogenous nitrate exposure. Plant foods and vegetables as well as processed foods contain nitrates/nitrites naturally and as an additive (65). This can be due to the homogeneity of the participants (TUMS employees) in our study. A study of 158 female omnivores and vegetarians aged 18–40 years showed that in vegetarians compared to omnivores, plasma concentrations of KYN were significantly lower (66). In this study, as in our study, the results showed that consuming plant foods and not consuming animal foods leads to a decrease in the production of metabolites. Furthermore, the results of studies have shown that healthier diets, such as the Mediterranean diet, were associated with lower levels of KYN (67). The results of the studies showed that vegetarians may have a better liver condition compared to omnivores. Since more than 95% of Trp in the diet is metabolized in the hepatic KYN pathway, therefore, in people with less animal food intake and more plant consumption, a better living condition can accelerate this pathway and thus lead to lower levels of all KYN (55). In the present study, we evaluated the amount of nitrate and nitrite intake from plant sources, but we could not measure the amount of intake of water as one of the most important sources of nitrate and nitrite. This can be an important reason for not finding significant relationships for some results. The use of FFQ, a validated 144-item questionnaire that depends on the memory of our study subjects, may prevent finding a significant relationship. Moreover, the metabolites were measured in the serum, but their measurement in the feces could also help make the results more significant. As well as, environmental and biological factors and elements such as temperature and weather conditions, soil composition, light, type and amount of nitrogen fertilizer consumption, crop cultivation, and harvest period, cause significant changes in nitrate and nitrite amounts (68, 69). As a result, the consumption of products from different regions with different conditions can be very effective on our results.

## 5 Strengths and limitations

As far as we know, this is the first study to investigate the association between dietary nitrates/nitrites intake and the gut microbial metabolites TMAO and KYN in adults. The use of a valid baseline FFQ to assess regular dietary intake and determine nitrate/nitrite intake was the main strength of the present study. It is the first study that examines the intake of nitrate and nitrite from two plant and animal sources and determines both the amount of total intake and intake by source. In addition, in this study, a wide range of confounding factors has been considered to achieve reliable results.

The cross-sectional design of the research is considered one of the main limitations, and due to the cross-sectional nature of the data, it cannot be used to infer causality. Next, although a large number of confounders were controlled in this study, we cannot deny the existence of residual confounders. The size of the sample and the homogeneity of the participants can also be considered another limitation of the present study.

## 6 Conclusion

The present evidence demonstrated there is a significant relationship between the intake of animal sources of nitrate and nitrite and the increase in the gut microbial metabolite TMAO and KYN levels. High intake of animal sources of nitrate and nitrite can increase the odds of having the highest levels of TMAO and KYN. Further clinical trials and additional prospective and cohort studies are needed to better evaluate and understand the potential effects. Moreover, these studies can assess the safe and toxic doses of nitrate and nitrite consumption.

## Data availability statement

The original contributions presented in the study are included in the article/supplementary material, further inquiries can be directed to the corresponding author.

## Ethics statement

The studies involving humans were approved by TUMS evaluated and approved the study protocol (IR.TUMS.MEDICINE.REC.1401.064). The studies were conducted in accordance with the local legislation and institutional requirements. The participants provided their written informed consent to participate in this study. Written informed consent was obtained from the individual(s) for the publication of any potentially identifiable images or data included in this article.

## Author contributions

AM: Conceptualization, Writing – original draft. MM: Project administration, Visualization, Writing – review & editing. FA: Writing – review & editing, Data curation, Formal analysis, Software. BB: Investigation, Writing – review & editing. AD: Writing – review & editing. PK: Writing – review & editing. ZR: Writing – review & editing, Visualization. KM: Visualization, Writing – review & editing, Funding acquisition.
